# Functional adaptation to mechanical loading in both cortical and cancellous bone is controlled locally and is confined to the loaded bones

**DOI:** 10.1016/j.bone.2009.08.054

**Published:** 2010-02

**Authors:** Toshihiro Sugiyama, Joanna S. Price, Lance E. Lanyon

**Affiliations:** Department of Veterinary Basic Sciences, The Royal Veterinary College, University of London, Royal College Street, London NW1 0TU, UK

**Keywords:** Mechanical loading, Functional adaptation, Local regulation, Physiological strains, Mouse

## Abstract

In order to validate whether bones' functional adaptation to mechanical loading is a local phenomenon, we randomly assigned 21 female C57BL/6 mice at 19 weeks of age to one of three equal numbered groups. All groups were treated with isoflurane anesthesia three times a week for 2 weeks (approximately 7 min/day). During each anaesthetic period, the right tibiae/fibulae in the DYNAMIC + STATIC group were subjected to a peak dynamic load of 11.5 N (40 cycles with 10-s intervals between cycles) superimposed upon a static “pre-load” of 2.0 N. This total load of 13.5 N engendered peak longitudinal strains of approximately 1400 microstrain on the medial surface of the tibia at a middle/proximal site. The right tibiae/fibulae in the STATIC group received the static “pre-load” alone while the NOLOAD group received no artificial loading. After 2 weeks, the animals were sacrificed and both tibiae, fibulae, femora, ulnae and radii analyzed by three-dimensional high-resolution (5 μm) micro-computed tomography (μCT). In the DYNAMIC + STATIC group, the proximal trabecular percent bone volume and cortical bone volume at the proximal and middle levels of the right tibiae as well as the cortical bone volume at the middle level of the right fibulae were markedly greater than the left. In contrast, the left bones in the DYNAMIC + STATIC group showed no differences compared to the left or right bones in the NOLOAD or STATIC group. These μCT data were confirmed by two-dimensional examination of fluorochrome labels in bone sections which showed the predominantly woven nature of the new bone formed in the loaded bones. We conclude that the adaptive response in both cortical and trabecular regions of bones subjected to short periods of dynamic loading, even when this response is sufficiently vigorous to stimulate woven bone formation, is confined to the loaded bones and does not involve changes in other bones that are adjacent, contra-lateral or remote to them.

## Introduction

Since Frost's introduction of the concept of the “mechanostat” [1], it has been accepted that bone mass and architecture are regulated in response to the local strains engendered in their tissue by functional loading. This mechanism has been the subject of a number of *in vivo* studies in animals in which artificial loads have been applied to the bones on one side and the modelling and remodelling responses in the loaded bones compared with those in the non-loaded contra-lateral pair [2–29]. For this approach to be valid, it is necessary to be sure that the adaptive response of the loaded bones is confined to those bones and does not influence their contra-lateral controls. This assumption has been challenged by the work of Sample et al. [30] who recently reported that in rapidly growing male rats a single period of dynamic high-magnitude axial loading of the ulna on one side was associated with significant levels of new cortical bone formation at the periosteal surface of the contra-lateral non-loaded ulna and in the cortical regions of adjacent bones in the loaded limbs. These responses were prevented by neuronal blockade. The authors [30] inferred from this that mechanically adaptive bone (re)modelling is controlled by processes with substantial systemic and central nervous components. If this inference were true, the focus of research into the mechanisms of mechanically adaptive bone (re)modelling would need to shift away from local responses and toward systemic and central regulation.

Although their inference did not accord with our experience [31], we could find no published studies specifically directed to establishing that the (re)modelling of bones contra-lateral to those which had been loaded was not different from that in bones in comparable animals where no bones had received artificial loading. Since use of the contra-lateral non-loaded limb as a control has become accepted practice, we undertook the present study to assess whether this was indeed the case. C57BL/6 mice are extensively used as the background of genetically modified animals in the field of bone research, and therefore we used the C57BL/6 mouse unilateral tibia/fibula axial loading model [12,27,29]. This model has the advantage over the ulna loading model [2,8,30] of enabling the study of trabecular as well as cortical bone compartments.

## Materials and Methods

### Animals

Virgin, female C57BL/6 mice at 8 weeks of age were purchased from Charles River Laboratories, Inc. (Margate, UK) and group-housed in sterilized polypropylene cages with free access to water and a maintenance diet containing 0.73% calcium, 0.52% phosphorus and 3.5 IU/g vitamin D (RM1; Special Diet Services Ltd., Witham, UK) in a 12:12-h light/dark cycle, with room temperature at 21 ± 2 °C. Body weight was measured once a week until sacrifice at 21 weeks of age. All procedures complied with the UK Animals (Scientific Procedures) Act 1986 and were reviewed and approved by the ethics committee of the Royal Veterinary College (London, UK).

### Experimental design

At 19 weeks of age, 21 mice were randomly allocated to three equal numbered groups. In the NOLOAD group, neither left nor right tibiae/fibulae received any artificial load. In the STATIC group, the right tibia/fibula received a small (2.0 N) static “pre-load” whose primary purpose is to hold the bone in the cups during loading. In the DYNAMIC + STATIC group, a larger (11.5 N) dynamic load was superimposed upon the 2.0-N static “pre-load”. Except for these differences in the loading regimen, all three groups received the same treatment. This included isoflurane-induced anesthesia for three alternate days a week for 2 weeks (approximately 7 min/day) during which loading took place. Normal cage activity was allowed between the treatments. High doses of calcein (50 mg/kg; Sigma Chemical Co., St. Louis, MO) and alizarin (50 mg/kg; Sigma Chemical Co.) were injected intraperitoneally on the first and last days of the treatments (days 1 and 12), respectively. At 21 weeks of age (day 15), the mice were euthanized and their tibiae, fibulae, femora, ulnae and radii were collected for analysis.

### In vivo external mechanical loading

The apparatus and protocol for dynamically loading the mouse tibia/fibula have been reported previously [12,13,27,29,32]. In brief, the flexed knee and ankle joints are positioned in concave cups; the upper cup, into which the knee is positioned, is attached to the actuator arm of a servo-hydraulic loading machine (Model HC10; Zwick Testing Machines Ltd., Leominster, UK) and the lower cup to a dynamic load cell. The tibia/fibula is held in place by a low level of continuous static “pre-load”, onto which is superimposed higher levels of intermittent “dynamic” load.

In the present study, 2.0 N was used as the static “pre-load” which was held for 400 s according to the original protocol [12]. The 11.5 N of “dynamic” load was superimposed onto the 2.0-N static “pre-load” in a series of 40 trapezoidal-shaped pulses (0.025 s loading, 0.050 s hold at 13.5 N and 0.025 s unloading) with a 10-s rest interval between each pulse. Strain gauges attached to the medial surface of the tibial shaft of similar 19-week-old female C57BL/6 mice showed that at a proximal/middle site (37% of the bone's length from its proximal end) a peak load of 13.5 N engendered approximately 1400 microstrain [29].

Although a peak load of 12.0 N can induce significant osteogenic responses in both cortical and trabecular bone [27], we selected a higher peak load (13.5 N) which was sufficient to induce woven bone formation in the loaded tibia [29]. Woven bone is generally seen in areas where the strain-related stimulus is high. Sample et al. [30] reported that it was at the “high” level of peak load that dynamic loading of the ulna resulted in (re)modelling responses in other bones that were not loaded. By using a loading regimen that stimulated woven bone formation, we sought to provide a stringent test for the presence of regional or systemic influences on mechanically adaptive (re)modelling in bones other than those being loaded.

### High-resolution micro-computed tomography (μCT) analysis

**Fig. 1:**
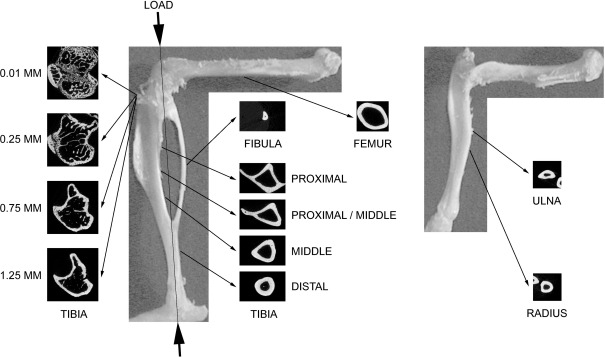
Direction of mechanical loading in the tibia/fibula and representative transverse μCT images at the analyzed sites in the tibia, fibula, femur, ulna and radius of a 21-week-old female C57BL/6 mouse. In the tibia, proximal (25% of the bone's length from its proximal end), proximal/middle (37%), middle (50%) and distal (75%) sites in cortical bone and two sites 0.01–0.25 mm (containing primary spongiosa) and 0.25–1.25 mm (secondary spongiosa) distal to growth plate in trabecular bone were analyzed. In the fibula, femur, ulna and radius, the middle (50%) sites in cortical bones were analyzed.

The tibiae, fibulae, femora, ulnae and radii from both sides in each animal were collected after sacrifice, stored in 70% ethanol and scanned by μCT (SkyScan 1172; SkyScan, Kontich, Belgium) with a pixel size of 5 μm. The images of the whole bones were reconstructed by the SkyScan software. As shown in Fig. 1, three-dimensional structural analyses were performed by the SkyScan software for the following regions:

(1) 0.5-mm-long sections at proximal (25% of the bones' length from their proximal ends), proximal/middle (37%), middle (50%) and distal (75%) sites in cortical bone of the tibiae;
(2) two sites 0.01–0.25 mm (containing primary spongiosa) and 0.25–1.25 mm (secondary spongiosa) distal to the growth plate in trabecular bone of the proximal tibiae; and
(3) 0.5-mm-long sections at the middle (50%) site in cortical regions of the fibulae, femora, ulnae and radii.

The parameters evaluated included periosteally enclosed volume, bone volume and medullary volume in the regions of cortical bone and percent bone volume (bone volume/tissue volume), trabecular number and trabecular thickness in the trabecular regions.

### Calcein and alizarin labels imaging by confocal microscopy

After scanning by μCT, the bones were dehydrated, cleared and embedded in methyl methacrylate as previously described [33]. Transverse segments were obtained by cutting with an annular diamond saw. Images of calcein and alizarin-labelled bone sections were visualized using the argon 488-nm laser and the HeNe 543-nm laser, respectively, of a confocal laser scanning microscope (LSM 510; Carl Zeiss MicroImaging GmbH, Jena, Germany) at similar regions as the μCT analysis. In the cortical regions, periosteal and endosteal labels and inter-label bone areas were measured as newly formed bone area at each region and normalized by total cortical bone area using ImageJ software (version 1.42; http://rsbweb.nih.gov/ij/) [30].

### Statistical analysis

All data are shown as mean ± SE. Body weight was compared by one-way ANOVA. In the analysis of bones, the left and right sides in each group were compared by paired *t*-test, and then those in all three groups by one-way ANOVA followed by a post hoc Bonferroni or Dunnett T3 test. Statistical analysis was performed using SPSS for Windows (version 17.0; SPSS Inc., Chicago, IL), and *p* < 0.05 was considered as significant.

## Results

### Body weight and bone lengths

As shown in Tables 1 and 2, there were no statistically significant differences in body weight or longitudinal lengths of the tibiae, fibulae, femora, ulnae and radii.

### Tibiae

**Fig. 2:**
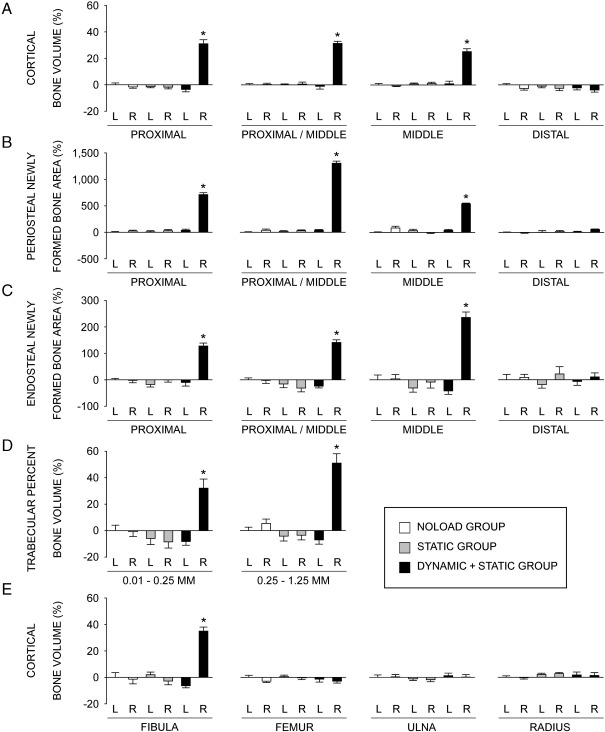
Relative values, analyzed by μCT and histomorphometry, of the left and right bones in the NOLOAD, STATIC and DYNAMIC + STATIC groups compared to the left bones in the NOLOAD group. L = left, R = right. (A) Cortical bone volume analyzed by μCT at the proximal (25% of the bone's length from its proximal end), proximal/middle (37%), middle (50%) and distal (75%) sites of the tibia. (B) Periosteal labels and inter-label bone area, analyzed by histomorphometry, normalized by total cortical bone area at the proximal, proximal/middle, middle and distal sites of the tibia. (C) Endosteal labels and inter-label bone area, analyzed by histomorphometry, normalized by total cortical bone area at the proximal, proximal/middle, middle and distal sites of the tibia. (D) Trabecular percent bone volume analyzed by μCT at two sites 0.01–0.25 mm (containing primary spongiosa) and 0.25–1.25 mm (secondary spongiosa) distal to the growth plate in the proximal tibia. (E) Cortical bone volume analyzed by μCT at the middle (50%) site of the fibula, femur, ulna and radius. Data are the mean ± SE (*n* = 6–7). ⁎*p* < 0.05 versus all other five values by one-way ANOVA followed by a post hoc Bonferroni or Dunnett T3 test.

Analysis by μCT showed that in the cortical regions of the tibiae in the DYNAMIC + STATIC group, periosteally enclosed and cortical bone volumes in the right loaded side were markedly higher than those of the contra-lateral non-loaded side at the proximal (+15.5 ± 1.0% and +35.9 ± 3.2%, respectively; *p* < 0.01), proximal/middle (+18.8 ± 0.6% and +32.7 ± 1.6%, respectively; *p* < 0.01) and middle (+13.3 ± 2.2% and +24.0 ± 2.2%, respectively; *p* < 0.01) sites (Table 3; Fig. 2A). There were no significant differences at the distal site. Medullary volume in the cortical region of the right loaded tibiae was smaller compared to that of the left tibiae at the proximal site (− 10.2 ± 2.8%; *p* < 0.01). In contrast to these differences between loaded and non-loaded bones in the DYNAMIC + STATIC group, there were no significant differences in the periosteally enclosed bone volume, cortical bone volume or medullary volume between the left and right tibiae in the STATIC or NOLOAD group. There were also no differences in these cortical parameters among the non-loaded tibiae in any group (Table 3; Fig. 2A).

**Fig. 3:**
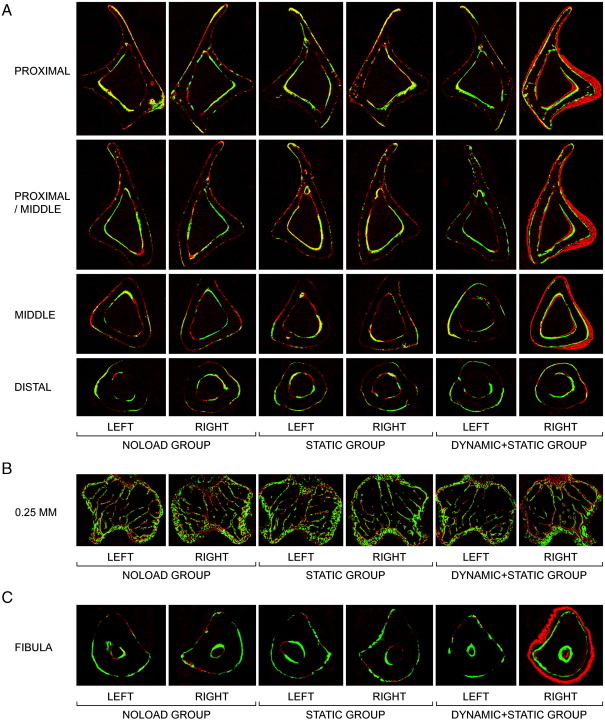
Representative transverse fluorochrome-labelled images. (A) Cortical bone at the proximal (25% of the bone's length from its proximal end), proximal/middle (37%), middle (50%) and distal (75%) sites of the tibia. (B) Trabecular bone at the site 0.25 mm distal to the growth plate in the proximal tibia. (C) Cortical bone at the middle (50%) site of the fibula. Green: calcein label injected on the first day of loading (day 1). Red: alizarin label injected on the last day of loading (day 12).

In the fluorochrome-labelled images, woven bone was clearly present at the proximal, proximal/middle and middle, but not distal, sites in the right loaded tibiae of the DYNAMIC + STATIC group (Fig. 3A). No woven bone formation was observed in the non-loaded tibiae in any group. Histomorphometry confirmed the marked increases in both periosteal and endosteal bone formation of the right loaded tibiae in the DYNAMIC + STATIC group and the absence of such new bone formation in the non-loaded tibiae (Table 3; Figs. 2B and 2C). This analysis detected a small but significant increase in periosteal bone formation at the distal site of the right loaded tibia in the DYNAMIC + STATIC group that was not revealed by μCT (Table 3).

In trabecular bone of the proximal tibia in the DYNAMIC + STATIC group, the right loaded side had markedly higher percent bone volume, trabecular number and trabecular thickness (0.01–0.25 mm site: +44.5 ± 7.6% [*p* < 0.01], + 18.0 ± 4.2% [*p* = 0.03], and + 21.0 ± 3.9% [*p* < 0.01], respectively; 0.25–1.25 mm site: + 62.5 ± 7.6%, + 27.8 ± 6.4%, and + 26.3 ± 1.7%, respectively [*p* < 0.01]) compared to the left non-loaded side (Table 4; Fig. 2D). In contrast, no differences in these parameters were observed between the left and right proximal tibiae in the STATIC or NOLOAD group. Furthermore, there were no significant differences between the left non-loaded tibiae of the DYNAMIC + STATIC group and left or right tibiae of the STATIC or NOLOAD group. Fluorochrome-labelled images confirmed these μCT results (Fig. 3B). The only difference detected other than in the right loaded tibiae of the DYNAMIC + STATIC group was decreased trabecular thickness at the 0.01- to 0.25-mm site in the right loaded tibiae of the STATIC group compared to the left tibiae in the NOLOAD group (− 6.8 ± 0.9%; *p* < 0.01) (Table 4).

### Fibulae

In cortical bone of the middle fibula in the DYNAMIC + STATIC group, periosteally enclosed and cortical bone volumes in the right loaded side were markedly higher (+ 36.9 ± 3.3% and + 44.1 ± 3.2%, respectively; *p* < 0.01) than those of the contra-lateral non-loaded side (Table 5; Fig. 2E). In contrast, no differences in these parameters were detected among the non-loaded fibulae in all groups. Fluorochrome-labelled images confirmed a marked increase in periosteal bone formation of the right loaded fibulae in the DYNAMIC + STATIC group and no difference in bone formation between the left non-loaded fibulae in the DYNAMIC + STATIC group and the left or right fibulae in the STATIC or NOLOAD group (Fig. 3C).

### Femora, ulnae and radii

The data for the femora, ulnae and radii are shown in Table 5 and Fig. 2E. In the DYNAMIC + STATIC group as well as the STATIC and NOLOAD groups, there were no differences in periosteally enclosed and cortical bone volumes in the cortical regions between the left and right femora, ulnae and radii. The fluorochrome-labelled images confirmed the lack of difference in periosteal bone formation among these bones (data not shown).

## Discussion

The experiments reported here were designed to establish whether, in studies where bones in one limb are loaded artificially, it is valid to assume that the (re)modelling in the contra-lateral bones is uninfluenced by such loading, thus allowing them to be used as non-loaded controls. The data presented support the validity of the assumption, in the C57BL/6 mouse unilateral tibia/fibula axial loading model [12,27,29] at least, since they showed no difference in bone (re)modelling between the bones of appropriately matched mice in which no bones were loaded and those contra-lateral to bones which had received static or static plus dynamic loading. From this we draw the narrow inference that bones in the contra-lateral limbs to those loaded at physiological levels sufficient to stimulate a vigorous osteogenic response can be used as non-loaded controls. We also draw the wider inference that functionally adaptive control of bone architecture is a local phenomenon within each bone that does not involve adjacent, regional or contra-lateral bones. The lack of uniformity in response in different regions of the loaded tibia suggests that the domain in which local strains influence (re)modelling is not only confined to the loaded bone but also is regional within it. While we have no reason to believe that this inference does not have general applicability, prudence dictates that it should be verified in each experimental situation where it is employed.

Our present experiment was not designed to establish the potential involvement of the nervous system in bones' functionally adaptive response. In the earliest experiments using artificial loading, Hert et al. [34] showed that adaptation took place in the tibia when the sciatic nerve had been sectioned. This accords with our experience [13]. Functional adaptation to loading has also been shown not to be affected by pharmacological blockade of the sympathetic nervous system [22]. These findings give us no reason to suggest that it is necessary to invoke nervous control in order to explain bones' functionally adaptive control of bone (re)modelling.

It was also not our intention to reproduce the experimental conditions in Sample et al.'s [30] study nor to explore experimentally the inconsistencies between their data and ours. There are a number of ways in which loading of one bone can have substantial effects on (re)modelling of adjacent and remote bones that are independent of normal, strain-related functionally adaptive (re)modelling. For example, new bone formation may be stimulated by the effects of trauma or interference with blood supply or be associated with the repair processes which any follow these events. We have no way of assessing whether these may have contributed to the responses reported by Sample et al. The animals they used were rapidly growing male Sprague–Dawley rats and young growing bone is more sensitive to such effects. The loading regimen they employed in the ulna was a severe one based on one of our earlier protocols [2] (3750 microstrain; 1500 cycles at 4 Hz [a total of 375 s]). In contrast, the loading regimens we use in the tibia/fibula as well as ulna to assess strain-related adaptation (less than 2000 microstrain; 40 cycles at 10 Hz with 10-s intervals between each cycle [a total of 400 s]) [12,13,27,29] are designed to produce a realistic physiological stimulus capable of stimulating a measurable osteogenic response while avoiding collateral stimulation associated with trauma and interference with blood supply both within the bone and around the loading cups. We select to use “three-dimensional” high-resolution (5 μm) μCT rather than “two-dimensional” fluorescent histomorphometry as our main tool to quantify functional adaptation in order to be able to analyze precisely comparative sites of the small mouse loaded and contra-lateral non-loaded bones. In our present study, when we employed the same histomorphometric analysis as Sample et al. [30], it revealed no substantial differences from the μCT data and thus confirmed the absence of any differences in (re)modelling between non-loaded bones regardless of whether they were contra-lateral to bones which had been loaded or to those which had not.

Our inference that strain-related functional adaptation in bone is a local phenomenon that does not extend to other bones or involve systemic or nervous intervention is limited to strains within the physiological range. Strains higher than this, or those repeated far more often, or perhaps with faster strain rates may well induce damage in the bone tissue and/or damage-related changes in the bone cells. In this situation, it is quite possible that the responses to these events may spread beyond the bones actually loaded and incorporate systemic involvement and/or involvement of the nervous system. Indeed, Sample et al. [30] observed no or less systemic and contra-lateral (re)modelling responses when they employed lower strains (760 and 2000 microstrains). The immediate experimental implication of this is that it would be prudent in any study that relies on use of contra-lateral non-loaded bones as controls to establish the level of loading-related stimulation that does not exceed the level necessary to stimulate local, strain-related functional adaptation. More intensive strain regimens may engender effects that extend beyond the local confines of the loaded bones. The wider implication may be that there is a distinction between the mechanisms involved in strain-related functional adaptation, the (re)modelling of which leads to adaptive changes in bone architecture presumably to regulate functional strains and the trauma-related (re)modelling which involves wider responses.

In the present study, a static load of 2.0 N did not affect cortical bone of the right loaded tibiae/fibulae or their longitudinal lengths. However, the right loaded proximal tibiae in the STATIC group had decreased trabecular thickness at the site 0.01–0.25 mm distal to the growth plate compared to the same site in the left proximal tibiae in the NOLOAD group. Since short periods of a higher level of static load can suppress bone formation [35], the current static “pre-load” of 2.0 N we used should be reduced in future studies nearer to the static “pre-load” of 0.2 N employed by Fritton et al. [14].

In conclusion, the data presented here, obtained from skeletally mature female C57BL/6 mice, suggest that the (re)modelling response of bones subject to short periods of artificial loading that engenders physiological strains is confined to the bones that are loaded. There is no reason to believe that this is a unique feature of these mice or the specifics of the tibia/fibula axial loading model [12,27,29]. The narrow implication of these findings is that since loading of one bone at physiological levels does not influence (re)modelling in bones that are contra-lateral, adjacent or remote to the bones that are loaded, the contra-lateral bones can be used as non-loaded controls. However, this should be established for each experimental model. The wider implication of this finding is that the mechanisms for physiological, strain-related, functional adaptation can legitimately be examined as local phenomena. In contrast, it is clear that, when the intensity of a strain regimen increases, the responses to it may extend to include a far wider spectrum of influences.

## Figures and Tables

**Table 1:** Body weight in female C57BL/6 mice that received no artificial loading, static (2.0 N) loading or static (2.0 N) plus dynamic (11.5 N) loading in the right tibia/fibula for 2 weeks.

	NOLOAD	STATIC	DYNAMIC + STATIC
19 weeks old (g)	22.7 ± 0.2	23.3 ± 0.2	23.3 ± 0.5
21 weeks old (g)	23.2 ± 0.3	23.4 ± 0.3	23.6 ± 0.6

Mean ± SE (*n* = 7). No significant differences between the NOLOAD, STATIC and DYNAMIC + STATIC groups.

**Table 2:** Longitudinal lengths of the tibia, fibula, femur, ulna and radius in 21-week-old female C57BL/6 mice that received no artificial loading, static (2.0 N) loading or static (2.0 N) plus dynamic (11.5 N) loading in the right tibia/fibula for 2 weeks.

	NOLOAD left	NOLOAD right	STATIC left	STATIC right	DYNAMIC + STATIC left	DYNAMIC + STATIC right
Tibia (mm)	18.4 ± 0.1	18.3 ± 0.1	18.3 ± 0.1	18.3 ± 0.1	18.4 ± 0.1	18.4 ± 0.1
Fibula (mm)	10.1 ± 0.1	10.0 ± 0.1	10.1 ± 0.1	10.1 ± 0.1	10.2 ± 0.1	10.1 ± 0.1
Femur (mm)	16.4 ± 0.1	16.5 ± 0.1	16.4 ± 0.1	16.5 ± 0.1	16.4 ± 0.1	16.3 ± 0.1
Ulna (mm)	13.8 ± 0.1	13.8 ± 0.1	13.7 ± 0.1	13.8 ± 0.1	13.7 ± 0.1	13.8 ± 0.1
Radius (mm)	11.2 ± 0.1	11.2 ± 0.1	11.2 ± 0.1	11.2 ± 0.1	11.2 ± 0.1	11.2 ± 0.1

Mean ± SE (*n* = 7). No significant differences between left and right in the NOLOAD, STATIC and DYNAMIC + STATIC groups.

**Table 3:** Cortical parameters analyzed by μCT and histomorphometry in the tibia of 21-week-old female C57BL/6 mice that received no artificial loading, static (2.0 N) loading or static (2.0 N) plus dynamic (11.5 N) loading in the right tibia/fibula for 2 weeks.

	NOLOAD left	NOLOAD right	STATIC left	STATIC right	DYNAMIC + STATIC left	DYNAMIC + STATIC right
*Proximal*						
Periosteally enclosed volume (mm^3^)	0.719 ± 0.012	0.711 ± 0.009	0.715 ± 0.005	0.717 ± 0.010	0.720 ± 0.013	0.832 ± 0.007⁎
Bone volume (mm^3^)	0.418 ± 0.006	0.411 ± 0.004	0.412 ± 0.003	0.411 ± 0.005	0.403 ± 0.008	0.548 ± 0.013⁎
Medullary volume (mm^3^)	0.301 ± 0.007	0.300 ± 0.007	0.303 ± 0.004	0.306 ± 0.009	0.316 ± 0.009	0.284 ± 0.009⁎
Periosteal newly formed bone area (%)	7.7 ± 1.1	9.5 ± 1.3	8.5 ± 1.5	10.0 ± 1.6	10.6 ± 1.6	62.1 ± 2.7⁎
Endosteal newly formed bone area (%)	10.0 ± 0.6	9.8 ± 0.9	8.3 ± 1.0	9.9 ± 0.8	9.0 ± 1.4	22.8 ± 1.1⁎

*Proximal/middle*						
Periosteally enclosed volume (mm^3^)	0.609 ± 0.006	0.621 ± 0.005	0.620 ± 0.007	0.623 ± 0.011	0.624 ± 0.011	0.742 ± 0.004⁎
Bone volume (mm^3^)	0.382 ± 0.004	0.383 ± 0.003	0.382 ± 0.002	0.384 ± 0.006	0.378 ± 0.008	0.501 ± 0.006⁎
Medullary volume (mm^3^)	0.227 ± 0.005	0.238 ± 0.006	0.237 ± 0.007	0.238 ± 0.006	0.246 ± 0.006	0.240 ± 0.005
Periosteal newly formed bone area (%)	4.6 ± 0.4	6.4 ± 1.0	5.4 ± 0.6	6.0 ± 0.7	6.5 ± 0.5	64.3 ± 1.8⁎
Endosteal newly formed bone area (%)	9.4 ± 0.7	9.2 ± 1.1	7.9 ± 1.4	6.5 ± 1.3	7.2 ± 0.6	22.8 ± 0.9⁎

*Middle*						
Periosteally enclosed volume (mm^3^)	0.504 ± 0.006	0.499 ± 0.006	0.508 ± 0.007	0.507 ± 0.008	0.506 ± 0.012	0.573 ± 0.011⁎
Bone volume (mm^3^)	0.307 ± 0.003	0.305 ± 0.001	0.309 ± 0.002	0.310 ± 0.003	0.310 ± 0.006	0.384 ± 0.007⁎
Medullary volume (mm^3^)	0.197 ± 0.004	0.194 ± 0.006	0.199 ± 0.005	0.197 ± 0.006	0.196 ± 0.008	0.189 ± 0.006
Periosteal newly formed bone area (%)	6.9 ± 0.8	12.8 ± 2.3	9.3 ± 1.8	6.8 ± 1.3	9.7 ± 1.2	44.5 ± 0.9⁎
Endosteal newly formed bone area (%)	5.5 ± 1.0	5.7 ± 0.9	3.8 ± 0.9	5.0 ± 1.2	3.2 ± 0.7	18.3 ± 1.1⁎

*Distal*						
Periosteally enclosed volume (mm^3^)	0.385 ± 0.004	0.378 ± 0.005	0.382 ± 0.005	0.379 ± 0.008	0.385 ± 0.006	0.389 ± 0.007
Bone volume (mm^3^)	0.281 ± 0.003	0.273 ± 0.003	0.278 ± 0.003	0.274 ± 0.005	0.275 ± 0.004	0.270 ± 0.004
Medullary volume (mm^3^)	0.103 ± 0.003	0.104 ± 0.003	0.104 ± 0.003	0.105 ± 0.003	0.110 ± 0.005	0.119 ± 0.006
Periosteal newly formed bone area (%)	8.9 ± 0.7	8.4 ± 1.0	9.2 ± 2.8	10.5 ± 1.6	9.7 ± 1.1	13.9 ± 0.8⁎
Endosteal newly formed bone area (%)	6.5 ± 1.3	7.0 ± 0.7	5.3 ± 0.9	7.9 ± 1.8	6.0 ± 1.0	7.1 ± 1.0

Mean ± SE (*n* = 6–7).

⁎ *p* < 0.05 versus left in the DYNAMIC + STATIC group by paired *t*-test.

**Table 4:** Trabecular parameters analyzed by μCT in the proximal tibia of 21-week-old female C57BL/6 mice that received no artificial loading, static (2.0 N) loading or static (2.0 N) plus dynamic (11.5 N) loading in the right tibia/fibula for 2 weeks.

	NOLOAD left	NOLOAD right	STATIC left	STATIC right	DYNAMIC + STATIC left	DYNAMIC + STATIC right
*0.01*–*0.25 mm*						
Percent bone volume (%)	15.2 ± 0.6	15.1 ± 0.6	14.3 ± 0.7	13.9 ± 0.7	13.9 ± 0.4	20.1 ± 1.1⁎
Trabecular number (mm^−^ ^1^)	3.19 ± 0.12	3.27 ± 0.12	3.17 ± 0.15	3.15 ± 0.18	2.99 ± 0.14	3.53 ± 0.13⁎
Trabecular thickness (μm)	47.6 ± 0.5	46.1 ± 0.5	45.2 ± 0.9	44.4 ± 0.4^#^	47.0 ± 1.4	56.9 ± 1.8⁎

*0.25*–*1.25 mm*						
Percent bone volume (%)	9.2 ± 0.2	9.7 ± 0.3	8.8 ± 0.3	8.9 ± 0.3	8.6 ± 0.3	13.9 ± 0.7⁎
Trabecular number (mm^−^ ^1^)	1.70 ± 0.06	1.77 ± 0.07	1.68 ± 0.07	1.73 ± 0.07	1.63 ± 0.07	2.08 ± 0.10⁎
Trabecular thickness (μm)	54.4 ± 0.5	54.8 ± 0.6	52.7 ± 0.7	51.4 ± 0.7	52.9 ± 1.5	66.8 ± 0.9⁎

Mean ± SE (*n* = 7).

⁎ *p* < 0.05 versus left in the DYNAMIC + STATIC group by paired *t*-test. ^#^*p* < 0.05 versus left in the NOLOAD group by one-way ANOVA followed by a post hoc Dunnett T3 test.

**Table 5:** Cortical parameters analyzed by μCT at middle site in the fibula, femur, ulna and radius of 21-week-old female C57BL/6 mice that received no artificial loading, static (2.0 N) loading or static (2.0 N) plus dynamic (11.5 N) loading in the right tibia/fibula for 2 weeks.

	NOLOAD left	NOLOAD right	STATIC left	STATIC right	DYNAMIC + STATIC left	DYNAMIC + STATIC right
*Fibula*						
Periosteally enclosed volume (mm^3^)	0.052 ± 0.002	0.052 ± 0.002	0.054 ± 0.002	0.051 ± 0.002	0.051 ± 0.001	0.070 ± 0.002⁎
Bone volume (mm^3^)	0.049 ± 0.002	0.049 ± 0.002	0.050 ± 0.001	0.048 ± 0.001	0.046 ± 0.001	0.067 ± 0.002⁎

*Femur*						
Periosteally enclosed volume (mm^3^)	0.849 ± 0.015	0.849 ± 0.009	0.863 ± 0.007	0.866 ± 0.012	0.847 ± 0.014	0.867 ± 0.012
Bone volume (mm^3^)	0.412 ± 0.007	0.400 ± 0.004	0.414 ± 0.004	0.411 ± 0.006	0.407 ± 0.009	0.400 ± 0.006

*Ulna*						
Periosteally enclosed volume (mm^3^)	0.145 ± 0.004	0.150 ± 0.004	0.149 ± 0.003	0.150 ± 0.004	0.154 ± 0.005	0.153 ± 0.005
Bone volume (mm^3^)	0.129 ± 0.002	0.129 ± 0.002	0.127 ± 0.002	0.127 ± 0.002	0.131 ± 0.003	0.129 ± 0.002

*Radius*						
Periosteally enclosed volume (mm^3^)	0.153 ± 0.003	0.152 ± 0.003	0.156 ± 0.002	0.157 ± 0.003	0.156 ± 0.003	0.155 ± 0.002
Bone volume (mm^3^)	0.119 ± 0.001	0.119 ± 0.001	0.122 ± 0.001	0.122 ± 0.001	0.121 ± 0.003	0.121 ± 0.003

Mean ± SE (*n* = 7).

⁎ *p* < 0.05 versus left in the DYNAMIC + STATIC group by paired *t*-test.
